# Transition to universal primary health care coverage in Brazil: Analysis of uptake and expansion patterns of Brazil’s Family Health Strategy (1998-2012)

**DOI:** 10.1371/journal.pone.0201723

**Published:** 2018-08-10

**Authors:** Monica Viegas Andrade, Augusto Quaresma Coelho, Mauro Xavier Neto, Lucas Resende de Carvalho, Rifat Atun, Marcia C. Castro

**Affiliations:** 1 Center for Development and Regional Planning, Federal University of Minas Gerais, Belo Horizonte, Minas Gerais, Brazil; 2 Takemi Program in International Health, Harvard T.H. Chan School of Public Health, Boston, Massachusetts, United States of America; 3 Faculty of Medical Sciences, University of São Paulo, São Paulo, São Paulo, Brazil; 4 Department of Global Health and Population, Harvard T.H. Chan School of Public Health, Boston, Massachusetts, United States of America; Netherlands Institute for Health Services Research, NETHERLANDS

## Abstract

Family Health Strategy, the primary health care program in Brazil, has been scaled up throughout the country, but its expansion has been heterogeneous across municipalities. We investigate if there are unique municipal characteristics that can explain the timing of uptake and the pattern of expansion of the Family Health Strategy from years 1998 to 2012. We categorized municipalities in six groups based on the relative speed of the Family Health Strategy uptake and the pattern of Family Health Strategy coverage expansion. We assembled data for 11 indicators for years 2000 and 2010, for 5,507 municipalities, and assessed differences in indicators across the six groups, which we mapped to examine spatial heterogeneities. Important factors differentiating early and late adopters of the Family Health Strategy were supply of doctors and population density. Sustained coverage expansion was related mainly to population size, marginal benefits of the program and doctors’ supply. The uptake was widespread nationwide with no distinct patterns among regions, but highly heterogeneous at the state and municipal level. The Brazilian experience of expanding primary health care offers three lessons in relation to factors influencing diffusion of primary health care. First, the funding mechanism is critical for program implementation, and must be accompanied by ways to support the supply of primary care physicians in low density areas. Second, in more developed and bigger areas the main challenge is lack of incentives to pursue universal coverage, especially due to the availability of private insurance. Third, population size is a crucial element to guarantee coverage sustainability over time.

## Introduction

Expansion of high quality primary health care (PHC) is a critical and necessary first step towards achieving Universal Health Coverage (UHC), one of the United Nations Sustainable Development Goals (SDG) [1]. Yet, PHC is far from universal and adequately provided worldwide [2–4]. In Brazil, the Family Health Strategy (FHS), created in 1994, is the mechanism of primary health care (PHC) delivery through the public system and henceforth the main platform for achieving universal health coverage (UHC) [5]. The Brazilian Health System comprises a mixed system in which around 75% of the whole population receives healthcare only through the public system (Unified Health System, SUS in Portuguese), while 25% has private insurance coverage. Even though part of population receives care through private insurance, these individuals also have the right to receive healthcare through the SUS, as there is no opting out in Brazil. In this context, UHC is only guaranteed by the public system. In the private system, PHC is not organized and has been driven by spontaneous demand. This model not only induces demand but is harmful for individuals since they do not receive longitudinal care.

Therefore, the FHS has a fundamental role to consolidate UHC in the Brazilian health system. As it is organized, the FHS operates as the opening gate to the SUS, guaranteeing that all individuals are registered and followed in the healthcare system. The FHS is a community-based program designed to provide integrated healthcare, in which family doctors play a key role. Its implementation is a responsibility of the mayor, and care is delivered through a Family Health Team (FHT) in a defined geographical area that has a population of about 4,000 people. Family Health Teams are based at Health Units, and are composed of a family physician, a nurse, a nursing assistant, and 4 to 12 community health agents (CHAs). CHAs provide comprehensive primary care support including managing chronic diseases, screening uptake immunization and healthy pregnancy, scheduling time at health units, and collecting socioeconomic information; they are expected to visit each household once per month, regardless of need.

All members of FHTs are hired and paid by the municipalities. The financing scheme of the FHS, created in 1998, uses a framework of incentives, as established by *Piso de Atenção Básica* (PAB), with two components: a fixed amount from the federal government (based on the number of inhabitants, to finance primary care expenses), and a variable amount conditioned on number of FHT. All money transfers from the federal government to municipalities are conditioned on the performance of municipalities in the management of primary health care that is monitored through information systems and regulation mechanisms.

Currently, the FHS covers 63% of the Brazilian population (about 122 million people), and provides evidence that scaling up PHC to achieve UHC in a large country with marked inequalities is feasible. Longitudinal analysis of the FHS uptake and expansion across municipalities from years 1998 to 2012 indicated positive associations with small population size, low population density, low coverage of private health insurance, low level of economic development, alignment of the political party of the mayor and the state governor, and the availability of healthcare personnel. Most importantly, the FHS uptake and expansion presented a heterogeneous pattern across municipalities [6].

In this paper we investigate if there are unique characteristics of municipalities that are associated with the timing of uptake and the pattern of diffusion of the FHS from years 1998 to 2012. Based on the speed and pattern of program diffusion, we group municipalities into six categories, and assess the spatial distribution of municipalities in each of these categories. We summarize social, economic, and demographic conditions for the municipalities in each category, and discuss the implications of these findings for further scale-up of the FHS.

## Materials and methods

The main outcome, proportion of the population covered by the FHS, was obtained from the Brazilian Ministry of Health [7]. As data are available monthly, July (mid-year period) was used as the temporal reference to assemble an annual time series, by municipality, from years 1998 (first year for which data are available) to 2012. Although the FHS was launched in 1994, the financial mechanisms that allowed money transfer from the federal government to municipalities were established only in 1998 [8]. Accordingly, in 1998 only 4.4% of the population was covered by the FHS. Thus using 1998 as the starting year of this analysis is unlikely to introduce bias. In addition, to address the creation of 58 new municipalities between 1998 and 2012, all data were assembled using the 1998 administrative division as a reference, consisting of 5,507 municipalities.

**Fig 1:**
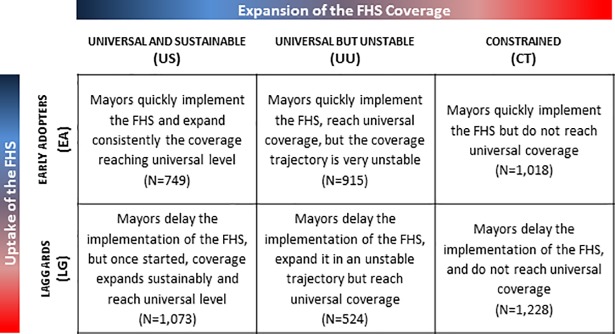
Proposed typology to describe the FHS uptake and coverage expansion in Brazil over 15 years (1998–2012). Criteria to classify municipalities in each of the six categories included: US–consistently expanded coverage, with only one negative annual growth rate of coverage in the 15-year period, reaching universal (> 95%) coverage in 2012; UU–expanded in an unstable trajectory, with more than one negative annual growth rate of coverage in the 15-year period, but reached universal (> 95%) coverage in 2012; CT–did not reach universal coverage in 2012; EA–implemented the FHS until 2000; and LG–implemented the FHS after 2000.

To characterize the transition to universal primary health care coverage we used two domains: timing of the FHS uptake, and pattern of the FHS coverage expansion (Fig 1). Timing of uptake assumed two possible groups: (i) early adopters (EA)–municipalities that implemented the FHS until the year 2000; and (ii) laggards (LG)–municipalities that implemented the FHS after 2000. The pattern of coverage expansion assumed three possible groups: (i) universal and sustainable (US)–municipalities that consistently expanded coverage, with only one negative annual growth rate of coverage over the 15-year period, and that reached universal coverage in 2012; (ii) universal and unstable (UU)–municipalities that had more than one negative annual growth rate of coverage in the 15-year period, but reached universal coverage in 2012; (iii) constrained (CT)–municipalities that did not reach universal coverage in 2012.

The categorization of these groups considers the fact that, for achieving sustained universal health coverage, it is not just the level of coverage that matters but how sustainable coverage remains over time. This is a critical issue for small municipalities where the loss of one family health team may represent moving from universal to no coverage. Also, there could be small fluctuations due to population growth; thus, universal coverage was defined as more than 95% of the population covered by the FHS. Combining the two domains, a municipality could be categorized in one of the six types, moving from best to worst scenario of speed of uptake and pattern of coverage combinations: (i) EA-US, (ii) EA-UU, (iii) EA-CT, (iv) LG-US, (v) LG-UU, and (vi) LG-CT.

### Data

Information on 11 indicators that summarize four domains–economic development, health conditions, supply of health care services, and size of the municipality–was collected for each municipality for the years 2000 and 2010 (years when Population Censuses were conducted in Brazil, and therefore when comprehensive data for municipalities are available). These four domains capture major factors affecting mayor´s ability and incentives to implement and expand FHS coverage in the municipality. The supply of health care services is related to the mayor´s ability to organize healthcare services including infrastructure, health equipments, and health professionals´ attraction. As the Brazilian health system is mixed, it is important to consider the presence of private health insurance, since they may compete for patients, and for providers or professionals. Economic development is associated to municipal network of healthcare services, and also to individual´s purchasing power and level of education. Health conditions are a proxy for population healthcare services demand. Finally, size of the municipality is related to the technology of providing PHC through FHS. As the FHS is a community-based program delivered in a well-defined geographic area, its implementation is directly associated to size and distribution of municipal population. Economic development included four indicators: municipal gross domestic product (GDP)–nominal GDP was deflated using the implicit price deflator [9, 10]; percentage of the population living in poverty (household income per capita under US$ 79.68 per month) [11]; Gini index–a measure of income inequality; and illiteracy rate for population aged more than 25 years [11]. Health conditions included two indicators: infant mortality rate, and percentage of population living in households with inadequate source of water and sewage treatment [11]; both indicators are commonly used as a proxy for living conditions [11]. Supply of health care services considered: number of doctors per 1,000 inhabitants [12, 13], proportion of the population with private health insurance (reliable data were not available prior to 2004) [14], and proportion of deaths with ill-defined cause (IDCD) [15]. IDCD are deaths categorized under Chapter XVIII of the 10^th^ revision of the International Statistical Classification of Diseases and Related Health Problems (ICD-10), containing only a description of symptoms and signs of diseases. The proportion of deaths with ill-defined cause is expected to capture the local capacity to organize healthcare services, the quality of medical assistance, problems in the access to health services, and population health status [16, 17]. Lastly, size of the municipality included both population size and population density [18, 19].

All data were extracted from publicly available sources, and are de-identified. This study was approved by the institutional review board of the Harvard T.H. Chan School of Public Health, Protocol # IRB16-0157.

### Statistical analysis

Considering timing of uptake and pattern of coverage expansion of the FHS for the period 1998 to 2012, each municipality was assigned to one of the six groups defined by the proposed categorization, and the results were mapped using Geographical Information System (GIS). To assess if the main source of spatial variation in the categorization was driven by uptake or by expansion of the FHS, we separately mapped municipalities by domains’ categories. All mapping was done in ArcGIS 10.2 (ESRI; Redlands, CA), utilizing map files obtained from the Brazilian Institute of Geography and Statistics (IBGE).

The 11 selected indicators were summarized for each of the six categories. A multivariate test of means, assuming heterogeneous covariance across groups, was used to assess differences of each indicator across the groups, but also across each separate category. All calculations were done in STATA v.12 (Stata Corp., College Station, TX, USA).

## Role of funding source

No funding.

## Results

**Fig 2:**
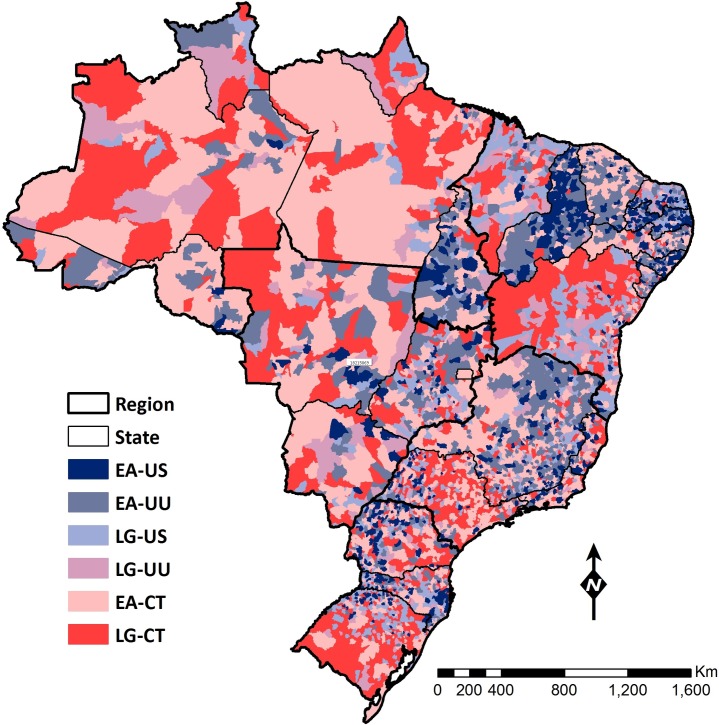
Municipal distribution of the typology. Color code for the six groups follows the color scheme shown in Fig 1. Shades of blue represent early adoption and faster expansion; shades of red indicate late adoption and constrained expansion.

Fig 1 indicates the number of municipalities that matched each of the six categories. Considering the nominal scale of the categorization, 13.6% (749/5,507) of the municipalities were in the most favorable group (EA-US), and 22.3% (1,228/5,507) were in the worst group (LG-CT). Fig 2 shows the geographical distribution of this categorization, highlighting the heterogeneity in uptake and expansion of the FHS across municipalities (S1 Fig shows the names of regions and states in Brazil). Overall, the most favorable categories occurred in the Northeast region, with the exception of the states of Bahia and Maranhão that presented, respectively, 36% and 19% of the municipalities in the worst category of LG-CT (S1 Table). In contrast, the North and Center-West regions, and Rio Grande do Sul presented with municipalities that fit the least favorable categories (S1 Table).

**Fig 3:**
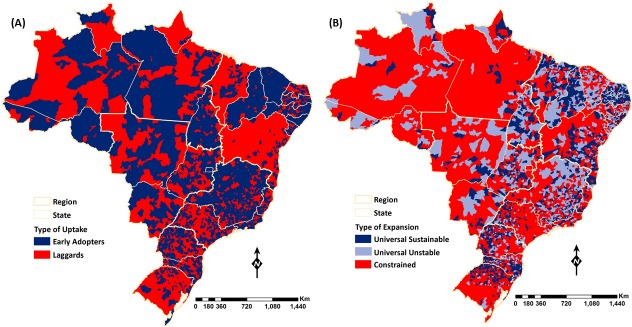
Municipal distribution of the typology considering the type of FHS uptake, and the type of FHS coverage expansion. Color code for the typology groups follows the color scheme shown in Fig 1. Shades of blue represent early adoption and faster expansion; shades of red indicate late adoption and constrained expansion.

Fig 3 displays the two domains of the categorization. Considering uptake, 48.7% (2,682/5,507) of the municipalities implemented the program before 2000 (EA), and together they represented 65% of the Brazilian population in 1998. The EA municipalities were located in all five regions and although within state variability was observed, four states (Rondônia, Alagoas, Ceará and Piauí) had 85% or more of the municipalities classified as EAs. In contrast, two states (Bahia and Maranhão) had 85% or more of the municipalities classified as LGs. Considering coverage expansion, Fig 3 highlights differences among regions: universal coverage was attained mostly in the Northeast region as well as in the states of Minas Gerais and Goiás, while constrained coverage was mostly observed in the North and Center-West regions. Eight states (Amazonas, Pará, Rondônia, Mato Grosso do Sul, Mato Grosso, Rio de Janeiro, São Paulo and Rio Grande do Sul) had 50% or more of the municipalities classified as CT.

**Fig 4:**
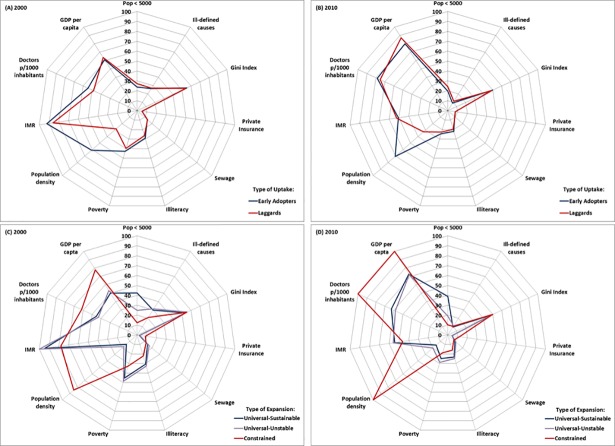
Selected municipal characteristics by the type of FHS uptake, and the type of FHS coverage expansion– 2000 and 2010. Data on access to private insurance refer to 2004 and 2010 (no reliable data available prior to 2004). Private insurance, poverty, illiteracy, Gini Index, population under 5,000, sewage, and ill-defined cause indicate percentages; all the remaining indicators were re-scaled to vary between 0–100. For all variables, a value of 100 represents the most favorable situation in both years taking into account the six groups of municipalities.

Regarding social, economic, demographic and health conditions of the municipalities, Fig 4 and S4 Table summarize the indicators by category domains. The means test for differences among categories is statistically significant for all indicators, suggesting that the categorization is adequate to differentiate the characteristics of municipalities regarding the uptake and expansion of the FHS. Considering the uptake domain, Fig 4A shows that the largest differences between municipalities classified as EAs or LGs in 2000 were driven by population density, number of doctors per 1,000 inhabitants, and infant mortality rate. In 2000, EA municipalities presented higher population density and higher supply of doctors per 1,000 inhabitants. This suggests that the supply of doctors may affect program uptake, and low population density could be a barrier to implementation. More favorable conditions with regards to illiteracy rate, percentage of population living in poverty, and Gini Index in 2000 (S2 Table), were often associated with LGs. Although these indicators improved for both EAs and LGs in 2010, statistically significant differences persisted. Considering the supply of health care services, between 2000 and 2010 there was a large reduction in the proportion of deaths with ill-defined cause, accompanied by an increase in the number of doctors per 1,000 inhabitants for both groups; in 2010 the proportion of deaths with ill-defined cause was smaller for EAs and significantly different from LGs (Fig 4B and S2 Table).

Considering the coverage expansion domain, Fig 4C shows the comparison among indicators for 2000. Municipalities classified as CT presented a higher level of economic development, better population health conditions, higher supply of health care, including private health insurance, and were bigger in 2000 –these characteristics were significantly different than the groups US and UU (S3 Table). These results suggest that CT municipalities presented comparatively better conditions to expand the FHS coverage, but failed to do so. Considering municipalities that reached universal coverage, UU and US, it is noticeable that those with a sustainable expansion of coverage during the period 1998–2012 were smaller, with lower level of economic development in 2000, and had a slightly higher supply of doctors. The major difference between UU and US groups was the percentage of municipalities with population lower than 5,000 inhabitants, indicating that population size is a fundamental factor to determine the type of FHS expansion. Also, even with less economic development, US municipalities presented a higher supply of doctors compared to UU. The comparison between 2000 and 2010 shows improvements in indicators related to economic development and health status (reduction in poverty, illiteracy, infant mortality rate, and in the proportion of deaths with ill-defined cause) for the three groups in the coverage expansion domain, while the gap in the number of doctors per 1,000 inhabitants across groups widened, with the largest gap observed between CT and UU (Fig 4D). Also, there was an increase in the proportion of the population covered by private health insurance in all three groups, and the gap among CT municipalities compared to US and UU widened: in 2010, the average proportion of private health insurance in CT municipalities was 0.12 while in US it was 0.04.

## Discussion

To the best of our knowledge, this is the first study in Brazil and worldwide that has examined the association between sub-national characteristics and the uptake and the consistency of coverage expansion of primary health care in a country. Municipalities were classified into one of six possible categories, based on their performance in the years 1998 to 2012. Our results showed that the categorization differentiated municipalities according to socioeconomic and healthcare supply characteristics. The main differences observed between EA and LG were population density and doctors´ supply, suggesting that economic development was not the main determinant of uptake. Conversely, for the expansion domain, CT municipalities presented better economic development, larger supply of healthcare, and were bigger with high population density, while UU and US were less developed, with higher health needs, and were smaller.

The FHS uptake was widespread across Brazil, without any discernible pattern among regions. At the state level, however, the pattern was distinct, indicating the importance of local policies that prioritized the FHS. There were no important differences regarding economic development between EAs and LGs in 2000, suggesting that funding mechanisms implemented by the national government provided enough financial incentives to program uptake. The most important factors differentiating LGs and EAs were supply of doctors and population density: in 1998, on average, EAs had 11% more doctors per 1,000 inhabitants than LGs, and EAs had a population density 117% greater than LGs. These results corroborate previous evidence showing that lack of primary care physicians is one of the most important barriers to FHS coverage expansion [20, 21]. In fact, it is important to note that there is not a shortage of physicians in Brazil, but an unequal distribution among regions [22].

Low population density was also an important barrier for the FHS uptake. These areas are more prone to have less access to health care due to harder difficulties to organize services, and consequently more challenges to implement the FHS. Recognizing these difficulties, in 2011 the Ministry of Health introduced different values of financial support conditioned on socioeconomic indicators such as: GDP per capita, percentage of the population receiving the conditional cash transfer (*Bolsa Família*), presence of private health insurance, percentage of population living in extreme poverty, and population density [23]. While the establishment of differentiated financial incentives taking into account the municipal characteristics was a step forward, the attraction and retention of physicians in rural/remote areas is one of the main challenges that needs to be addressed by the Brazilian government. In 2013, Brazil launched the More Doctors Program aimed at supplying doctors to underserved areas [24]; while its impact still needs to be evaluated, policies focused on medical training and/or doctor’s career path are likely to be more effective to guarantee supply of health care providers in these remote areas.

The sustainability of FHS coverage expansion is related mainly to three factors: population size, marginal social benefits of the program, and doctors’ supply. Social benefits of the program are conditioned upon the network of healthcare services previously available to population. In small municipalities, PHC is often the only source of health care; thus, stable FHS teams increase population welfare, but absence of the FHS may create unpopularity for Mayors who do not prioritize supply of these services. Small and more vulnerable municipalities, which perceive higher marginal social benefits of expanding and maintaining universal coverage of PHC, achieved better performance of FHS coverage expansion (universal and stable) during the period 1998–2012. In contrast, for bigger and more developed municipalities, where typically there are other sources of healthcare in addition to the FHS, program benefits are not so clearly appreciated by the population. Furthermore, management of the FHS may be easier in smaller municipalities given the small number of teams (as the number of FHTs is based on the population size, small municipalities may need just one or two teams to reach universal coverage of primary care). These considerations may help explain the high-performing (blue) areas observed in the Northeast region, and in the states of Goiás, Tocantins, and Minas Gerais (Fig 3). Limited supply of doctors, an important factor associated with constrained coverage of the FHS, is likely to explain the relative underperformance (red) of areas in the North region (Fig 3). In 2010 the average number of doctors per 1,000 inhabitants in the North region was 0.59, which is almost one fourth of the average observed for the whole country (2.15).

The Brazilian experience provides some lessons for the expansion of PHC in contexts of regional inequality and where a dual health system (private and public) operates. First, the funding mechanism is critical for the mayor´s decision to implement the strategy, and must be accompanied by mechanisms to support the supply of primary care physicians in low density areas. The Brazilian experience showed that financial incentives were effective to determine the uptake of the program as well as the consolidation of the FHS as a national policy, but they did not guarantee its sustainability and universal coverage. Second, population size is a crucial element to guarantee coverage stability over time; management of the program seems to be harder in bigger municipalities since the number of professionals is proportional to population size (the majority of municipalities that reached universal coverage of PHC through the FHS had less than 20,000 inhabitants). Third, in more developed and bigger municipalities the main challenge is not the lack of resources, but, instead, the lack of incentives to pursue universal coverage, given the availability of private health insurance. For example, in São Paulo, the most developed and populous state in Brazil, almost 40% of the population was covered by private health insurance in 2017 [14]. Yet, they have the right to use public services, and do so for high complexity services. Therefore, policy makers in those areas are much more focused in providing medium and high complexity healthcare. In this context, although the FHS has not achieved universality, it is a critical policy to overcome inequities in PHC access.

The main limitation of this paper concerns municipal indicators. Four different domains were included to characterize municipalities, and the choice of variables was guided by availability and reliability of data. Other variables related to supply of healthcare services and population health conditions could be elicited but were not available. Regarding FHS coverage, we used the definition proposed by the Ministry of Health. It is calculated relating population size with the number of FHS teams, and the common recommended number of people covered by each team. Thus, it does not consider the spatial distribution of the population in the municipality. As a result, isolated communities could be outside the catchment area of the teams. Nevertheless, the distortions are unlikely to be very large and unlikely to alter our conclusions.

## Supporting information

S1 Fig Regional and State Administrative Divisions of Brazil. The map shows the boundaries and names of each of the 27 states, and indicates in color the boundaries of the five regions of Brazil.
(PDF)

S1 Table Percentage distribution of municipalities in each state according to categorization domain. Each line of the table indicates the percentage of municipalities in the state that match categories of FHS uptake and expansion.
(PDF)

S2 Table Descriptive statistics of municipal characteristics considering the type of FHS uptake, 2000 and 2010. (PDF)

S3 Table Descriptive statistics of municipal characteristics considering the type of FHS coverage expansion, 2000 and 2010. (PDF)

S4 Table Descriptive statistics of municipal characteristics considering the categorization of the transition to universal primary health care coverage. (PDF)
