# Correction: Transition to universal primary health care coverage in Brazil: Analysis of uptake and expansion patterns of Brazil’s Family Health Strategy (1998–2012)

**DOI:** 10.1371/journal.pone.0251764

**Published:** 2021-05-11

**Authors:** Monica Viegas Andrade, Augusto Quaresma Coelho, Mauro Xavier Neto, Lucas Resende de Carvalho, Rifat Atun, Marcia C. Castro

The following information is missing from the Funding statement: Monica Viegas Andrade acknowledges Fapemig for financial support (PPM-00273-16). No additional external funding was received for this study.
