# Supplementary material for: Transition to universal primary health care coverage in Brazil: Analysis of uptake and expansion patterns of Brazil’s Family Health Strategy (1998-2012)
Source: PLoS One. 2018 Aug 10;13(8):e0201723. doi: 10.1371/journal.pone.0201723 (PMC6086633; doi:10.1371/journal.pone.0201723)

S1 Fig. Regional and State Administrative Divisions of Brazil.
The map shows the boundaries and names of each of the 27 states, and indicates in color the boundaries of the five regions of Brazil.

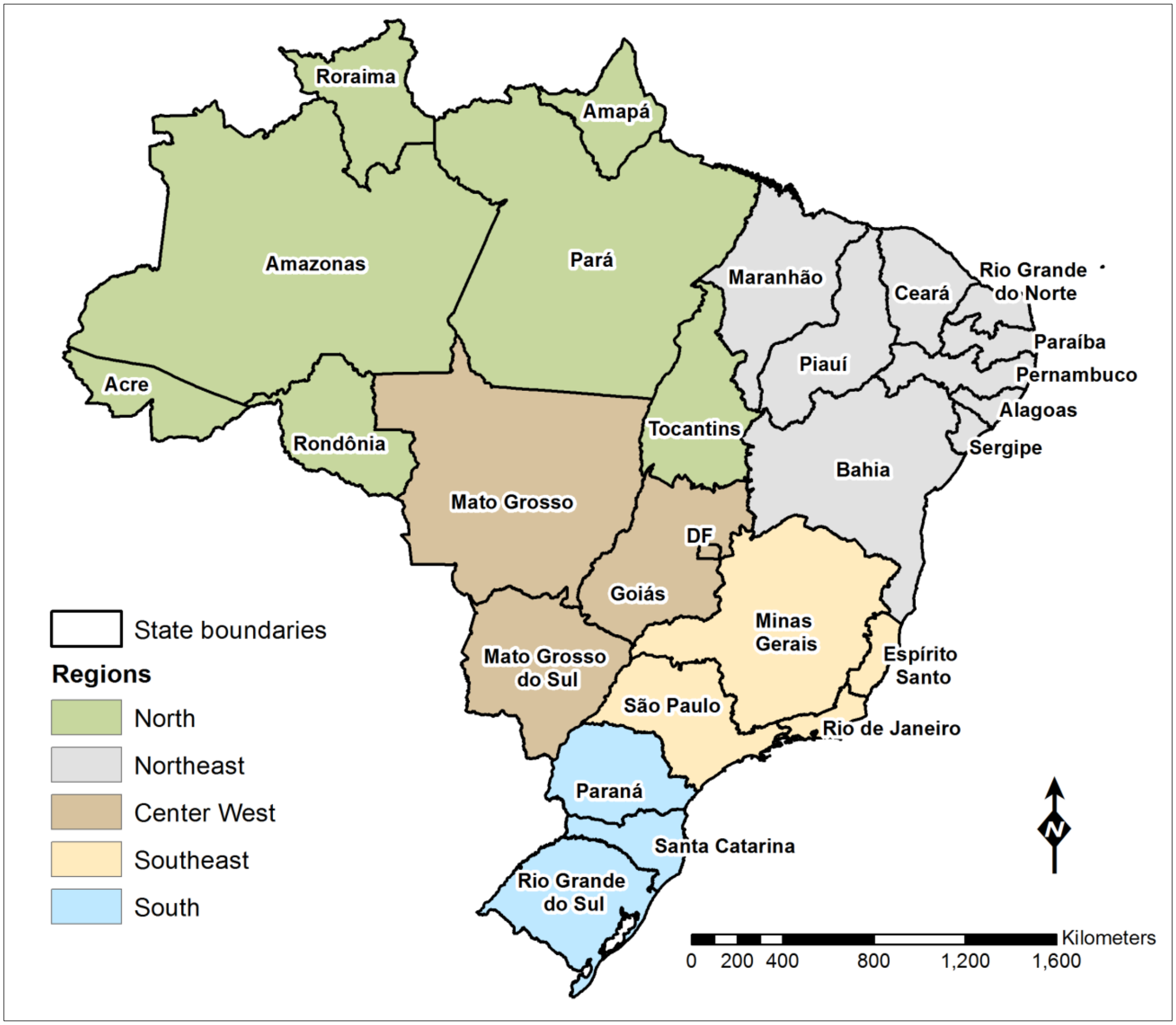

Supplement: S1 Fig — The map shows the boundaries and names of each of the 27 states, and indicates in color the boundaries of the five regions of Brazil. (PDF) [file pone.0201723.s001.pdf]
