# Supplementary material for: Transition to universal primary health care coverage in Brazil: Analysis of uptake and expansion patterns of Brazil’s Family Health Strategy (1998-2012)
Source: PLoS One. 2018 Aug 10;13(8):e0201723. doi: 10.1371/journal.pone.0201723 (PMC6086633; doi:10.1371/journal.pone.0201723)
Supplement: S1 Table — Each line of the table indicates the percentage of municipalities in the state that match categories of FHS uptake and expansion. (PDF) [file pone.0201723.s002.pdf]

S1 Table. Percentage distribution of municipalities in each state according to categorization domain. Each line of the table indicates the percentage of municipalities in the state that match categories of FHS uptake and expansion.

Regions		Uptake and Coverage Expansion						Uptake		Coverage expansion		
	States	EA-US	LG-US	EA-UU	LG-UU	EA-CT	LG-CT	LG	EA	US	UU	CT
North	AC (N=22)	0	13.64	45.45	4.55	18.18	18.18	36.36	63.64	13.64	50	36.36
	AM (N=62)	1.61	4.84	11.29	9.68	35.48	37.1	51.61	48.39	6.45	20.97	72.58
	AP (N=16)	0	25	0	25	12.5	37.5	87.5	12.5	25	25	50
	PA (N=143)	1.4	7.69	6.29	8.39	37.06	39.16	55.24	44.76	9.09	14.69	76.22
	RO (N=52)	5.77	0	30.77	0	57.69	5.77	5.77	94.23	5.77	30.77	63.46
	RR (N=15)	0	26.67	13.33	26.67	6.67	26.67	80	20	26.67	40	33.33
	TO (N=139)	19.42	17.27	30.94	7.91	19.42	5.04	30.22	69.78	36.69	38.85	24.46
Northeast	AL (N=101)	39.6	0.99	34.65	2.97	21.78	0	3.96	96.04	40.59	37.62	21.78
	BA (N=415)	2.65	31.33	3.37	19.52	6.51	36.63	87.47	12.53	33.98	22.89	43.13
	CE (N=184)	11.41	0	44.57	3.26	40.22	0.54	3.8	96.2	11.41	47.83	40.76
	MA (N=217)	1.84	42.4	7.37	23.5	5.53	19.35	85.25	14.75	44.24	30.88	24.88
	PB (N=223)	39.01	34.53	11.66	10.76	2.69	1.35	46.64	53.36	73.54	22.42	4.04
	PE (N=185)	10.81	8.65	32.43	4.86	37.3	5.95	19.46	80.54	19.46	37.3	43.24
	PI (N=221)	47.06	5.88	33.94	3.17	8.6	1.36	10.41	89.59	52.94	37.1	9.95
	RN (N=166)	19.28	36.75	16.27	19.88	3.01	4.82	61.45	38.55	56.02	36.14	7.83
	SE (N=75)	17.33	10.67	36	8	21.33	6.67	25.33	74.67	28	44	28
MidWest	GO (N=242)	12.81	26.45	18.18	11.16	14.05	17.36	54.96	45.04	39.26	29.34	31.4
	MS (N=77)	6.49	15.58	10.39	16.88	18.18	32.47	64.94	35.06	22.08	27.27	50.65
	MT (N=126)	8.73	7.14	19.84	10.32	29.37	24.6	42.06	57.94	15.87	30.16	53.97
	DF (N=1)	0	0	0	0	100	0	0	100	0	0	100
Southeast	ES (N=77)	22.08	10.39	9.09	10.39	23.38	24.68	45.45	54.55	32.47	19.48	48.05
	MG (N=853)	10.55	16.65	30.01	9.96	20.4	12.43	39.04	60.96	27.2	39.98	32.83
	RJ (N=91)	12.09	10.99	9.89	7.69	39.56	19.78	38.46	61.54	23.08	17.58	59.34
	SP (N=645)	7.91	11.63	5.12	4.96	21.71	48.68	65.27	34.73	19.53	10.08	70.39
South	PR (N=399)	18.55	17.79	9.77	6.77	21.55	25.56	50.13	49.87	36.34	16.54	47.12
	RS (N=467)	7.92	32.76	1.71	5.78	10.49	41.33	79.87	20.13	40.69	7.49	51.82
	SC (N=293)	19.45	27.99	12.63	9.22	13.65	17.06	54.27	45.73	47.44	21.84	30.72
