# Supplementary material for: Transition to universal primary health care coverage in Brazil: Analysis of uptake and expansion patterns of Brazil’s Family Health Strategy (1998-2012)
Source: PLoS One. 2018 Aug 10;13(8):e0201723. doi: 10.1371/journal.pone.0201723 (PMC6086633; doi:10.1371/journal.pone.0201723)
Supplement: S2 Table — (PDF) [file pone.0201723.s003.pdf]

S2 Table. Descriptive statistics of municipal characteristics considering the type of FHS uptake, 2000 and 2010

Selected Indicators/Year	Early Adopters (N=2,682)		Laggards (N=2,825)		Means test	
**2000**	**Mean**	**Std. Dev.**	**Mean**	**Std. Dev.**	**F-test**	**p-value**
Population density	128.84	685.15	59.22	239.39	24.81	p<0.001
Proportion of deaths with ill-defined cause	0.26	0.21	0.27	0.24	0.53	0.47
GDP per capita (R$)	27126.60	32202.07	28263.00	33070.68	1.67	0.20
Doctors per 1,000 inhabitants	1.52	1.41	1.34	1.14	24.18	p<0.001
Gini index	0.55	0.07	0.54	0.07	44.35	p<0.001
Percentage of households without piped water and sewage	13.74	14.94	13.87	16.32	0.09	0.77
Infant mortality rate	34.60	13.80	32.20	13.98	41.26	p<0.001
Illiterate rate (pop over 25 years old)	28.98	15.50	26.28	15.20	42.44	p<0.001
Percentage of individuals living in poverty	42.64	22.25	39.65	23.18	23.94	p<0.001
Private health insurance coverage (2004)	0.05	0.08	0.05	0.09	0.02	0.90
Percentage of municipalities with less than 5,000 inhabitants	0.238	0.426	0.238	0.445	8.55	p<0.001
**2010**	**Mean**	**Std. Dev.**	**Mean**	**Std. Dev.**	**F-test**	**p-value**
Population density	149.70	775.07	69.25	288.85	25.53	p<0.001
Proportion of deaths with ill-defined cause	0.09	0.09	0.11	0.11	67.97	p<0.001
GDP per capita (R$)	35636.14	42824.91	38822.35	45108.33	7.23	0.01
Doctors per 1,000 inhabitants	2.18	2.04	2.09	1.81	3.35	0.07
Gini index	0.50	0.06	0.49	0.07	28.51	p<0.001
Percentage of households without piped water and sewage	9.03	11.78	9.47	13.84	1.62	0.20
Infant mortality rate	19.62	6.96	18.98	7.31	11.09	p<0.001
Illiterate rate (pop over 25 years old)	21.74	13.05	19.51	12.41	42.06	p<0.001
Percentage of individuals living in poverty	24.20	17.37	22.42	18.43	13.60	p<0.001
Private health insurance coverage	0.08	0.11	0.08	0.11	0.06	0.81
Percentage of municipalities with less than 5,000 inhabitants	0.20	0.40	0.24	0.43	16.03	p<0.001
