# Supplementary material for: Transition to universal primary health care coverage in Brazil: Analysis of uptake and expansion patterns of Brazil’s Family Health Strategy (1998-2012)
Source: PLoS One. 2018 Aug 10;13(8):e0201723. doi: 10.1371/journal.pone.0201723 (PMC6086633; doi:10.1371/journal.pone.0201723)
Supplement: S3 Table — (PDF) [file pone.0201723.s004.pdf]

S3 Table. Descriptive statistics of municipal characteristics considering the type of FHS coverage expansion, 2000 and 2010

Selected Indicators/Year	US (N=1,822)		UU (N=1,439)		Constrained (N=2,246)		Means test	
**2000**	**Mean**	**Std. Dev.**	**Mean**	**Std. Dev.**	**Mean**	**Std. Dev.**	**Wald chi2**	**p-value**
Population density	30.37	45.81	37.42	111.87	179.74	783.09	85.57	p<0.001
Proportion of deaths with ill-defined cause	0.30	0.25	0.31	0.23	0.21	0.19	268.81	p<0.001
GDP per capita (R$)	22,279.04	19,556.87	23,612.84	40,387.18	34,739.63	34,288.86	215.59	p<0.001
Doctors per 1,000 inhabitants	1.26	0.91	1.19	1.04	1.71	1.59	165.88	p<0.001
Gini index	0.54	0.07	0.56	0.07	0.55	0.07	83.13	p<0.001
% households without piped water and sewage	14.12	15.01	16.07	15.41	12.09	16.14	55.92	p<0.001
Infant mortality rate	35.31	14.32	37.48	13.48	29.15	12.75	411.26	p<0.001
Illiterate rate (pop over 25 years old)	30.49	15.39	32.67	14.73	21.99	14.01	592.74	p<0.001
Percentage of individuals living in poverty	45.17	21.44	48.67	21.20	32.96	22.26	548.46	p<0.001
Private health insurance coverage (2004)	0.02	0.05	0.03	0.06	0.08	0.11	553.86	p<0.001
% municipalities with < 5,000 inhabitants	42.43	49.44	24.95	43.29	12.24	32.79	514.26	p<0.001
**2010**	**Mean**	**Std. Dev.**	**Mean**	**Std. Dev.**	**Mean**	**Std. Dev.**	**Wald chi2**	**p-value**
Population density	32.98	56.53	42.55	126.61	211.84	891.83	95.75	p<0.001
Proportion of deaths with ill-defined cause	0.09	0.10	0.11	0.11	0.10	0.10	12.15	p<0.001
GDP per capita (R$)	32,451.62	38,820.55	32,133.81	50,573.69	44,471.00	42,515.60	107.32	p<0.001
Doctors per 1,000 inhabitants	1.74	1.36	1.63	1.27	2.78	2.41	386.08	p<0.001
Gini index	0.48	0.06	0.50	0.06	0.50	0.07	68.69	p<0.001
% households without piped water and sewage	9.43	12.78	10.99	12.34	8.00	13.16	48.99	p<0.001
Infant mortality rate	20.37	7.81	20.93	6.97	17.37	6.19	322.02	p<0.001
Illiterate rate (pop over 25 years old)	23.21	12.90	24.65	12.42	15.87	11.32	612.70	p<0.001
Percentage of individuals living in poverty	24.78	17.37	28.74	17.24	18.58	17.65	317.03	p<0.001
Private health insurance coverage	0.04	0.07	0.05	0.08	0.12	0.14	570.21	p<0.001
% municipalities with < 5,000 inhabitants	38.91	48.77	19.04	39.28	10.11	30.15	488.50	p<0.001
