# Supplementary material for: Transition to universal primary health care coverage in Brazil: Analysis of uptake and expansion patterns of Brazil’s Family Health Strategy (1998-2012)
Source: PLoS One. 2018 Aug 10;13(8):e0201723. doi: 10.1371/journal.pone.0201723 (PMC6086633; doi:10.1371/journal.pone.0201723)
Supplement: S4 Table — (PDF) [file pone.0201723.s005.pdf]

S4 Table. Descriptive statistics of municipal characteristics considering the categorization of the transition to universal primary health care coverage

Selected indicators/year	EA-US (N=749)		EA-UU (N=915)		LG-US (N=1,073)		LG-UU (N=524)		EA-CT (N=1,018)		LG-CT (N=1,228)		Means Test	
	Mean	Std. Dev.	Mean	Std. Dev.	Mean	Std. Dev.	Mean	Std. Dev.	Mean	Std. Dev.	Mean	Std. Dev.	Wald chi2	p-value
Population density 2000	35.68	61.75	42.44	137.47	26.66	29.48	28.65	35.47	275.04	1,087.75	100.73	357.14	125.92	p<0.001
Population density 2010	39.79	78.48	48.82	155.48	28.23	32.79	31.61	40.48	321.25	1,228.77	121.15	430.83	139.73	p<0.001
Proportion of deaths with ill-defined cause 2000	0.30	0.24	0.30	0.22	0.30	0.25	0.34	0.25	0.21	0.18	0.21	0.20	274.95	p<0.001
Proportion of deaths with ill-defined cause 2010	0.08	0.08	0.10	0.10	0.11	0.11	0.12	0.12	0.09	0.08	0.11	0.10	101.60	p<0.001
GDP per capita 2000	21,638.19	20,601.99	23,594.69	39,609.82	22,726.39	18,789.76	23,644.53	41,748.27	34,339.28	30,429.55	35,071.53	37,194.91	219.85	p<0.001
GDP per capita 2010	31,400.00	48,174.07	31,070.76	45,009.38	33,185.69	30,648.92	33,990.09	59,047.71	42,856.37	34,971.04	45,809.51	47,850.20	114.31	p<0.001
Doctors per 1,000 inhabitants 2000	1.38	0.97	1.26	1.15	1.18	0.84	1.07	0.78	1.84	1.78	1.61	1.41	225.56	p<0.001
Doctors per 1,000 inhabitants 2010	1.75	1.33	1.64	1.32	1.74	1.39	1.60	1.19	2.99	2.65	2.60	2.18	389.65	p<0.001
Gini index 2000	0.54	0.07	0.56	0.07	0.53	0.07	0.55	0.07	0.56	0.06	0.54	0.07	130.48	p<0.001
Gini index 2010	0.49	0.06	0.50	0.06	0.48	0.64	0.50	0.06	0.51	0.06	0.49	0.07	102.61	p<0.001
% households without piped water and sewage 2000	13.12	14.00	15.60	14.71	14.80	15.64	16.88	16.55	12.51	15.65	11.73	16.54	62.67	p<0.001
% households without piped water and sewage 2010	8.33	11.06	10.60	11.88	10.21	13.82	11.67	13.09	8.13	12.07	7.89	14.00	60.24	p<0.001
Infant mortality rate 2000	36.25	14.12	37.66	13.41	34.66	14.42	37.18	13.61	30.64	12.96	27.92	12.43	452.45	p<0.001
Infant mortality rate 2010	20.84	7.85	20.73	6.61	20.04	7.77	21.26	7.54	17.72	6.10	17.08	6.25	331.41	p<0.001
Illiterate rate 2000 (pop > 25 years)	32.12	15.41	33.23	14.76	29.36	15.27	31.69	14.64	22.84	14.21	21.28	13.80	621.32	p<0.001
Illiterate rate 2010 (pop > 25 years)	24.61	13.17	25.17	12.58	22.24	12.61	23.73	12.09	16.53	11.62	32.06	11.03	640.98	p<0.001
Proportion of individuals living in poverty 2000	46.50	20.94	49.04	20.88	44.23	21.74	48.03	21.76	34.04	21.66	32.06	22.72	558.90	p<0.001
Proportion of individuals living in poverty 2010	25.36	16.73	28.84	16.87	24.38	17.80	28.57	17.87	19.18	16.96	18.09	18.18	320.23	p<0.001
Private health insurance coverage 2004	0.03	0.05	0.03	0.05	0.02	0.45	0.03	0.07	0.09	0.11	0.08	0.11	564.01	p<0.001
Private health insurance coverage 2010	0.04	0.07	0.05	0.08	0.04	0.07	0.05	0.08	0.12	0.13	0.12	0.14	575.41	p<0.001
Proportion of municipalities with < 5,000 inhabitants 2000	0.43	0.50	0.25	0.43	0.42	0.49	0.25	0.44	0.09	0.28	0.15	0.36	581.14	p<0.001
Proportion of municipalities with < 5,000 inhabitants 2010	0.38	0.49	0.19	0.39	0.40	0.49	0.19	0.39	0.07	0.25	0.13	0.33	549.25	p<0.001
